# Mirabile

**Published:** 1885-02

**Authors:** C. E. Francis


### MIRABILE!

BY C. E. FRANCIS, D. D. S.

Judging from much that is stated at society gatherings, printed in dental journals, or expressed in social interviews, it might sometimes be inferred that our specialty is honored with a certain but limited number of much favored individuals, upon whom dame fortune has lavished the most precious gifts from her store of coveted treasures. They seem born to luck—their star being ever in the ascendency—and a glow of infallibility is visibly and indelibly stamped o'er their unruffled brows. In their professional career all is as serene as a summer sky, and one's soul is nearly choked with envy as he reads or listens to recitals of brilliant achievements or incomparable successes. In the treatment of all cases that come within the range of their specialty they meet with wonderful results. Under the influence of their skill gold is manipulated with the most implicit obedience, and despite all adverse circumstances teeth loaded with the precious metal will withstand the ravages of all time. Their fillings of amalgam or tin are of like permanency. In the treatment of root canals they will penetrate the most tortuous and infinitesimal channels, and remove every atom of their contents. Their peculiar instruments search the very verge of the apical foramina but never pass a line beyond its limit, and fillings are carried thither so easily, so snugly, so nicely, and with such charming results that one can hardly help wishing that all his root cases could be transferred to their care. They can save pulps or fragments of pulps in all cases, and insure what there is left of them from any future trouble. They glory in cases of alveolar abscess, which invariably yields to their marvelous treatment. Pyorrhœa alveolaris succumbs to their magic touch, and teeth so loose as to wabble at every movement of the tongue are securely "pocketed" until they become as firm as a rock of granite. Cases of irregularity and artificial dentures give them no trouble; indeed, success is the crowning result of all their efforts.

What a pity that all of us could not be so highly favored.

